# Integrated health surveillance and early warning systems in China under the One Health perspective: progress and challenges

**DOI:** 10.1016/j.soh.2025.100132

**Published:** 2025-10-24

**Authors:** Zhichao Li, Dongliang Li, Jinwei Dong, Qixu Zhu, Youyi Zuo, Juan Pu, Lu Wang, Weipan Lei, Jun Cai, Qu Cheng, Yuzhe Li, Jing Yang, Yang Ju, Zhirui Wu

**Affiliations:** a Key Laboratory for Resource Use and Environmental Remediation, Institute of Geographic Sciences and Natural Resources Research, Chinese Academy of Sciences, Beijing 100101, China; b National Center for Translational Medicine, Shanghai Jiao Tong University, Shanghai 200240, China; c University of Chinese Academy of Sciences, Beijing 100049, China; d School of Geographical Sciences, University of Nottingham Ningbo China, Ningbo 315100, Zhejiang, China; e National Key Laboratory of Veterinary Public Health and Safety, Key Laboratory for Prevention and Control of Avian Influenza and Other Major Poultry Diseases, Ministry of Agriculture and Rural Affairs, College of Veterinary Medicine, China Agricultural University, Beijing 100193, China; f National Key Laboratory of Veterinary Public Health and Safety, College of Veterinary Medicine, China Agricultural University, Beijing 100193, China; g Key Laboratory for Biodiversity Science and Ecological Engineering, College of Life Sciences, Beijing Normal University, Beijing 100875, China; h Demonstration Center for Experimental Life Sciences & Biotechnology Education, College of Life Sciences, Beijing Normal University, Beijing 100875, China; i School of Public Health, Key Laboratory of Public Health Safety, Ministry of Education, Fudan University, Shanghai 200032, China; j Department of Epidemiology and Biostatistics, School of Public Health, Tongji Medical College, Huazhong University of Science and Technology, Wuhan 430030, Hubei, China; k Key Laboratory of Land Surface Pattern and Simulation, Institute of Geographical Sciences and Natural Resources Research, Chinese Academy of Sciences, Beijing 100101, China; l CAS Key Laboratory of Pathogen Microbiology and Immunology, Institute of Microbiology, Chinese Academy of Sciences, Beijing 100101, China; m School of Geography and Ocean Science, Nanjing University, Nanjing 210023, Jiangsu, China; n Global Health Research Center, Duke Kunshan University, Kunshan 215316, Jiangsu, China

**Keywords:** One Health, Surveillance system, Climate surveillance, Wildlife surveillance, Human health surveillance

## Abstract

Emerging infectious diseases (EIDs), whether newly identified or re-emerging in human and animal populations, pose significant threats to global public health. China has experienced multiple EIDs outbreaks in recent years, underscoring the need for robust surveillance and early warning systems. Although China has established surveillance systems for events affecting climate, wildlife, livestock and poultry, and humans, the current systems remain inadequate for the early detection, monitoring, and prevention of zoonotic spillover events. The “One Health” approach, which integrates human, animal, and environmental health, offers a comprehensive strategy for mitigating EIDs risks. This study reviews China's national-level surveillance and early warning systems from a “One Health” perspective, highlighting key limitations and proposing future directions to enhance preparedness and response capabilities. The findings are intended to inform policy improvements and strengthen interdisciplinary collaboration for effective EIDs management.

## 1 Introduction

Emerging infectious diseases (EIDs), either newly emergent or re-emergent in human or animal populations, have gained increasing global attention in recent years due to their substantial impact on public health [1]. China has experienced several significant EIDs that have profoundly affected public health and society, including the 2002–2003 Severe Acute Respiratory Syndrome (SARS) outbreak [2], the COVID-19 pandemic, highly pathogenic avian influenza (HPAI) H5N1 [3], and hand, foot, and mouth disease (HFMD) [4]. These disease outbreaks have underscored the critical need for early detection, surveillance and effective public health responses to prevent and control the occurrence and transmission of EIDs.

China has established nationwide disease surveillance systems that cover human, wildlife, and domestic animal populations. For example, the National Notifiable Diseases Surveillance System (NNDSS) collects and analyzes data on infectious diseases in human populations from healthcare facilities across the country [5,6]. The National Forestry and Grassland Administration (NFGA) is responsible for monitoring wildlife epidemics nationwide, and has established the national-level Wildlife Epidemic Disease Monitoring Terminal, comprising a network of monitoring stations spanning national, provincial, municipal, and county levels. Meanwhile, the Ministry of Agriculture and Rural Affairs (MARA) has built a nationwide monitoring network for animal diseases, targeting poultry (e.g., chickens, ducks, and geese), livestock (e.g., pigs, cows, and sheep), and special economic animals (e.g., minks and foxes). However, the current global climate and land use and land cover (LULC) changes are obvious. Environmental changes are altering the spatial and temporal interactions among wildlife/vectors, domestic animals, and humans, thereby increasing the risk of pathogen spillover [7,8]. For example, climate change and wetland reclamation are altering the distribution and migratory behaviors of wild birds, influencing their interactions with poultry in regions such as Poyang Lake in China [9]. In this context, the current surveillance systems remain limited in their capacity to detect, monitor, and prevent the emergence and early spread of novel pathogens [10,11].

The One Health approach aims to integrate human, animal, and environmental health to comprehensively monitor disease threats across species and ecosystems, and to propose coordinated prevention and control measures within an integrated environment–animal–human framework [12,13]. By combining data from environmental, animal, and human health domains, surveillance systems enhance the understanding of disease dynamics and improve the identification of emerging health risks [14]. In this context, this study reviews China's national-level surveillance and early warning systems from a One Health perspective and discusses current limitations and future implications.

## 2 Methods

### 2.1 Literature review

**Fig. 1:**
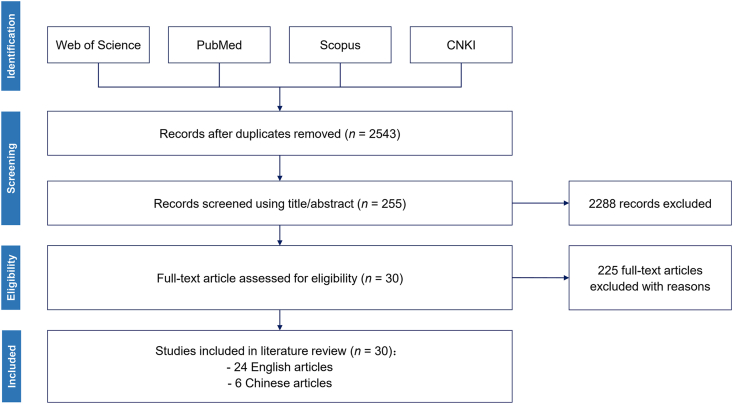
The workflow of the systematic search to retrieve articles following the PRISMA statement. Abbreviations: PRISMA, Preferred Reporting Items for Systematic Reviews and Meta-Analyses; CNKI, China National Knowledge Infrastructure.

This study used the Preferred Reporting Items for Systematic Reviews and Meta-Analyses (PRISMA) statement [15] to guide the literature process regarding national-level climate, animal, and human health surveillance systems (Fig. 1). Searches were conducted using the logical structure (meteorolog∗ OR livestock OR wild∗ OR “zoonotic disease” OR “infectious disease” OR epidemi∗) AND (“forecast∗ system” OR “monitor∗ system” OR “surveillance system” OR “response system” OR “information system” OR “early warning” OR “disease control”) AND (China OR Chinese) in PubMed, Web of Science, and Scopus databases. For Chinese literature, the logical structure: (meteorolog∗ OR livestock OR wild∗ OR “zoonotic disease” OR “infectious disease” OR epidemi∗) AND (“forecast∗ system” OR “monitor∗ system” OR “surveillance system” OR “response system” OR “information system” OR “early warning” OR “disease control”) AND (China OR Chinese) in Chinese was searched in the China National Knowledge Infrastructure (CNKI) database. All queries were limited to titles and abstracts. All records were merged, and duplicates were removed using the EndNote software. Then, we examined the title and abstract of each record to select relevant articles. Lastly, a full-text screening was conducted using the following criteria: (1) studies focused on China's surveillance or monitoring, including climate, ecosystem, wildlife, livestock, or human health; (2) studies presenting the relevant information on the national-level surveillance system.

### 2.2 Data analysis

We systematically selected 30 papers to assess China's national-level surveillance systems for climate, animal, and human health under the One Health approach (Table 1). These documents included 6 articles in Chinese and 24 in English. Among them, 9 articles were related to climate surveillance, 5 articles were related to animal surveillance, and 16 articles were related to human health surveillance. We manually extracted the information from each article, including the authors and publication date, title, surveillance system types, and language of the publication.

**Table 1:** National surveillance systems for climate, animal, and human health, as extracted from the selected articles.

ID	Authors and publication date	Article title	Surveillance system types	Language of the publication
1	Ji et al., 2023 [16]	China's public health initiatives for climate change adaptation	Climate	English
2	Shen et al., 2023 [17]	New version of the CMA-GFS dynamical core based on the predictor–Corrector time integration scheme	Climate	English
3	Sun et al., 2023 [18]	Multiscale feature analysis of forecast errors of 500 hPa geopotential height for the CMA-GFS model	Climate	English
4	Zhang et al., 2023 [19]	Key model technologies of CMA-GFS V4.0 and application to operational forecast	Climate	Chinese
5	Dong et al., 2024 [20]	Optimal assimilation of microwave upper-level sounding data in CMA-GFS	Climate	English
6	Liu and Zhong, 2021 [21]	Regional climate monitoring and assessment in the belt and road	Climate	English
7	Li et al., 2024 [22]	Influences of Graupel microphysics on CMA-GFS simulation of summer regional precipitation	Climate	English
8	Zhang et al., 2022 [23]	Interpretation of the IPCC AR6 on the impacts and risks of climate change	Climate	Chinese
9	Huang et al., 2023 [24]	From AR5 to AR6: Exploring research advancement in climate change based on scientific evidence from IPCC WGI reports	Climate	English
10	Wei et al., 2015 [25]	Biosecurity and disease management in China's animal agriculture sector	Animal	English
11	Peng et al., 2020 [26]	Construction of surveillance and prevention and control system for terrestrial wildlife-borne infectious diseases in China	Animal	Chinese
12	Qin et al., 2021 [27]	Wildlife-borne microorganisms and strategies to prevent and control emerging infectious diseases	Animal	English
13	Li et al., 2021 [28]	Wild animal and zoonotic disease risk management and regulation in China: Examining gaps and One Health opportunities in scope, mandates, and monitoring systems	Animal	English
14	Gao et al., 2023 [29]	Management of animal health data to inform policy in China	Animal	English
15	Zhang et al., 2017 [6]	Surveillance and early warning systems of infectious disease in China: From 2012 to 2014	Human	English
16	Ren et al., 2019 [5]	Systematic review: National notifiable infectious disease surveillance system in China	Human	English
17	Deng et al., 2023 [30]	Epidemiological characteristics of hemorrhagic fever of renal syndrome in China, 2004–2021	Human	Chinese
18	Qiu et al., 2023 [31]	The epidemiology of varicella and effectiveness of varicella vaccine in Ganyu, China: a long-term community surveillance study	Human	English
19	Yang et al., 2011 [32]	A nationwide web-based automated system for outbreak early detection and rapid response in China	Human	English
20	Yang et al., 2018 [33]	Comparing the similarity and difference of three influenza surveillance systems in China	Human	English
21	Wang et al., 2022 [34]	Epidemiology of influenza virus reinfection in Guangxi, China: a Retrospective analysis of a nine-year influenza surveillance data: Characteristics of influenza virus reinfection	Human	English
22	Kang et al., 2019 [35]	Comparing the timeliness of three types of influenza surveillance data in mainland China	Human	Chinese
23	Pan et al., 2024 [36]	Comparison of three influenza surveillance data sources for timely detection of epidemic onset—Chengdu city, sichuan province and Beijing Municipality, China, 2017–2023	Human	English
24	Sun et al., 2024 [37]	Drawing on the development experiences of infectious disease surveillance systems around the world	Human	English
25	Yang et al., 2017 [38]	China infectious diseases automated-alert and response system (CIDARS)	Human	English
26	Wang et al., 2017 [39]	How to select a proper early warning threshold to detect infectious disease outbreaks based on the China infectious disease automated alert and response system (CIDARS)	Human	English
27	Wang et al., 2017 [40]	‘Outbreak Gold standard’ selection to provide optimized threshold for infectious diseases early-alert based on China infectious disease automated-alert and response system	Human	English
28	Wang et al., 2019 [41]	Cognition on principle, daily operation, and early warning efficiency of CIDARS among online management professionals in Shanghai city	Human	Chinese
29	Lu et al., 2024 [42]	Developing a research network of early warning systems for infectious diseases transmission between China and Australia	Human	English
30	Peng and Yang, 2020 [43]	Early warning of epidemics: Towards a national intelligent syndromic surveillance system (NISSS) in China	Human	English

## 3 Results

### 3.1 Surveillance systems for meteorological factors and extreme weather

Climate surveillance focuses on climate variables (e.g., temperature, precipitation, humidity, wind speed, and greenhouse gas), as well as extreme weather events that increase health risks. Different regions of China face varying risks from extreme weather, including threats to water resources, ecosystems, and exposure to meteorological hazards, which in turn amplify climate-induced health risks: Northwest China faces accelerated snow and ice melt with increased flooding; North China experiences marked warming, drying, and water stress; Northeast China warms above the national average, with rising flood and permafrost risks; Southwest China suffers frequent winter–spring droughts and ecological stress; Central China sees alternating droughts and floods with wetland loss; and South China is increasingly exposed to extreme weather and seawater intrusion [16]. Such information is essential for predicting and mitigating the impacts of climate change on zoonoses such as dengue, plague, hemorrhagic fever with renal syndrome, and avian influenza in China.

The China Meteorological Administration (CMA) has developed a comprehensive operational numerical prediction system based on the Global/Regional Assimilation and Prediction System (GRAPES) model. This system includes the Global Forecast System (GFS) for 10-day deterministic forecast, the Global Ensemble Prediction System (GEPS) for 15-day ensemble forecast, the Mesoscale Model (MESO) for 36-h high-resolution forecast, the Regional Ensemble Prediction System (REPS) for 3-day regional ensemble prediction, and the Typhoon Model (TYM) for the Indian Ocean–Northwest Pacific 5-day typhoon forecast [17]. At the core of the system is the CMA-GFS (formerly GRAPES-GFS), a global model developed by the CMA Earth System Modeling and Forecasting Center [18,22]. The CMA-GFS comprises four major components: a dynamical framework based on the semi-implicit semi-Lagrangian (SISL) non-hydrostatic dynamic core; a modular package for physical processes; a four-dimensional variational data assimilation system; and global and regional assimilation and prediction systems [20]. CMA-GFS not only provides forecasts of global weather, precipitation, near-surface elements, and tropical cyclone path and intensity, but also provides initial and boundary conditions for limited regional mesoscale numerical prediction systems and various specialized model systems at national and regional centers. It also serves as a key data source and platform supporting the research, development, and application of artificial intelligence (AI) technologies in weather forecasting [19]. Table 2 shows the key features of CMA operational numerical prediction systems under the GRAPES framework. CMA plays a vital role in real-time meteorological operations, supporting high-frequency decision-making in emergency response and public services, and shares data with Belt and Road countries via the World Meteorological Organization Information System (WIS) [21].

**Table 2:** Key features of CMA operational numerical prediction systems under the GRAPES framework.

Models	Indicator types	Timeliness	Spatial distribution	Application
CMA-GFS	Weather, precipitation, near-surface elements, tropical cyclone path and intensity	10-day deterministic forecast	Global	Provides boundary conditions for regional systems; AI platform; emergency response support; WIS data sharing
CMA-GEPS	Probabilistic forecasts of multiple variables	15-day ensemble forecast	Global	Enhances uncertainty quantification and extreme event forecasting
CMA-MESO	Standard weather elements, mesoscale focus	China 36-h high-resolution forecast	Regional	City/province-level high-resolution forecasting
CMA-REPS	Probabilistic regional forecasts	China 3-day ensemble forecast	Regional	Supports short- to medium-range risk assessment
CMA-TYM	Typhoon path and intensity	Indian Ocean–Northwest pacific, 5-day forecast	Regional	Specialized typhoon early warning system

Abbreviations: CMA, China Meteorological Administration; GRAPES, Global/Regional Assimilation and Prediction System; GFS, Global Forecast System; GEPS, Global Ensemble Prediction System; MESO, Mesoscale Model; REPS, Regional Ensemble Prediction System; TYM, Typhoon Model.

China has also participated in the development of the Sixth Assessment Report of the Intergovernmental Panel on Climate Change (IPCC-AR6). The IPCC, positioned at the intersection of policy and science, plays a vital role in providing scientific evidence to inform climate action and solutions [24]. As a participating country in IPCC-AR6, China has not only contributed scientific strength to the global climate assessment, but also obtained key references from the report. IPCC-AR6 has deepened the systematic understanding of climate change risks and provided a scientific basis and methodological support for enhancing China's capabilities in climate risk monitoring, assessment, and adaptation [23]. In addition, China has actively participated in other IPCC activities, including the 62nd plenary session held in Hangzhou in February 2025, where the draft outline of the working group contributions and the methodology report for the Seventh Assessment Report (AR7) were discussed and adopted.

### 3.2 Surveillance systems for wild animals and livestock diseases

China is one of the countries with the greatest diversity and abundance of wild animals. Wild animals are natural or susceptible hosts for many infectious diseases. It is estimated that more than 1.2 million unknown virus species exist in China, and between 10,000 and 30,000 unknown bacteria may be present in wild mammals on the Qinghai–Xizang Plateau alone [27]. Wildlife surveillance refers to the systematic monitoring of the health status of wildlife populations, with particular emphasis on diseases that may pose a threat to human and domestic animal health. Surveillance covers a broad range of wildlife species, with a focus on those that may carry or transmit zoonotic diseases, such as bats, rodents, and birds. A robust wildlife disease surveillance infrastructure facilitates the comprehensive tracking of disease outbreaks in terrestrial wildlife populations and supports the early detection of potential zoonotic disease transmission. Since 2005, a total of 350 national terrestrial wildlife epidemic source and disease monitoring stations have been established in areas with high terrestrial wildlife concentrations in China, based on the National Animal Disease Control System Development Plan (2004–2008) [25]. The National Forestry and Grassland Administration has also established national wildlife disease and epidemic source monitoring stations in national-level nature reserves. In 2010, the former State Forestry Administration launched the Land Wildlife Epidemic Source Disease Monitoring and Prevention Information Direct Reporting System [26]. In 2013, the forestry department issued the *Management Measures for Monitoring and Control of Terrestrial Wildlife Epidemics and Epidemic Sources*, which outlines the procedures and responsibilities for monitoring, reporting, and managing terrestrial wildlife epidemics [28]. In 2014, in response to the development need for informatization, the direct reporting system was upgraded to a comprehensive management system, integrating and improving functions such as information collection, summarization and analysis, emergency response, and public education. This system was officially launched in 2017. As of now, 742 national terrestrial wildlife epidemic source disease monitoring stations, along with a large number of provincial (city and county)-level stations, have been established across the country. Together, they form a multi-level surveillance network centered on national stations and supported by provincial (city and county)-level stations [26]. The surveillance network is organized in a top-down manner, extending from the State Council to the NFGA, then to provincial and municipal forestry and grassland authorities, and ultimately to local monitoring stations. At the same time, surveillance information is reported in a bottom-up process, transmitted step by step from municipal and provincial authorities to the NFGA and the State Council. Horizontal collaboration mechanisms involve national and provincial information management centers, decision command centers, and relevant departments, working in conjunction with early warning and emergency support systems to provide timely support and feedback for rapid response and policy decision-making.

The Bureau of Animal Husbandry and Veterinary Services, a department under China's Ministry of Agriculture and Rural Affairs, is responsible for organizing national livestock surveillance and systematically monitoring the health status of poultry (e.g., chicken, duck, goose, pigeon, and quail) and livestock (e.g., pig, cattle, and sheep), with the aim of preventing, controlling, and eradicating animal diseases—particularly zoonotic diseases. China's poultry and livestock disease surveillance system plays a vital role in safeguarding animal health, ensuring food safety, and promoting agricultural sustainability. It is also a key component of the comprehensive zoonotic disease surveillance system. The system comprises national-, provincial-, city-, and county-level veterinary authorities, research institutes and diagnostic laboratories which oversee poultry and livestock breeding, compulsory vaccination, disease reporting, epidemiological investigations, and animal movement inspections [29]. The Bureau of Animal Husbandry and Veterinary Services under the MARA is responsible for national animal disease prevention and control, supported by four affiliated institutions: the China Animal Disease Control Center (CADC), the China Animal Health and Epidemiology Center (CAHEC), the China Institute of Veterinary Drug Control (IVDC), and the National Animal Husbandry Station. Together, these institutions manage national animal health data and provide technical support. CADC oversees the animal disease prevention information system and emergency command platform; CAHEC maintains the national epidemiology database and conducts early-warning analyses; IVDC supervises veterinary drug data; and the National Animal Husbandry Station focuses on animal husbandry and breeding. In addition, professional associations collect and analyze supplementary data on animal health and production, providing further input for policy and industry development [29]. Currently, China utilizes data management systems and information technologies to collect, analyze, and disseminate livestock disease surveillance data. This data-driven approach provides a scientific foundation for decision-making and supports the optimization of resources and prioritization of disease prevention and control efforts.

### 3.3 Surveillance systems for human diseases

China's surveillance system for human diseases is responsible for the continuous monitoring, collection, analysis, evaluation, and dissemination of information on the population health and disease trends using systematic methods. To support this, China has established the NNDSS, the country's most fundamental and long-standing infectious disease surveillance mechanism with the broadest coverage [6]. The NNDSS was developed in response to public health emergencies, and has evolved from a paper-based, hierarchical monthly reporting system to a real-time, internet-based reporting system launched in 2004. By 2013, the system covered all of the county-level centers for disease control and prevention (CDCs) in China [5]. A large volume of disease data generated by researchers from the China CDC has been reported through NNDSS [30,31].

After the outbreak of SARS in 2003, the Chinese government established the National Notifiable Infectious Diseases Reporting Information System (NIDRIS) in 2004. Based on the surveillance data from NIDRIS, the China Infectious Disease Automated-alert and Response System (CIDARS) was developed by the China CDC [32]. In recent years, three major surveillance systems—laboratory-confirmed case reporting, influenza-like illness (ILI) surveillance, and NIDRIS—have been used for influenza surveillance in China [33,35]. NIDRIS is characterized by wide coverage, mandatory reporting, high specificity, and real-time data transmission. The use of NIDRIS enhances the early detection of influenza epidemics, particularly when multiple respiratory pathogens are circulating simultaneously [36,34]. CIDARS was officially launched nationwide in 2008 [44,40]. It performs real-time daily analysis based on four key components: aberration detection, signal creation, signal distribution, and feedback response. The system automatically detects anomalies in data reported through NIDRIS and rapidly alerts the relevant county-level CDC via Short Message Service (SMS) [42]. Each day at 8 a.m., the CIDARS issues early warning signals regarding potential infectious disease outbreaks that occurred in the previous 24 h. Upon receiving a warning message, CDC staff are required to verify the signal promptly through telephone interviews or field investigations and to submit verification feedback within 12 h in CIDARS [39,41]. By 2011, CIDRAS covered 30 reportable infectious diseases. By 2015, it incorporated three early warning methods, the fixed-threshold detection method (FDM), a temporal model, and a spatiotemporal model, and had been implemented across all CDCs above the county level [38]. However, as data are primarily collected from medical institutions and data exchange between different departments remains limited, both NIDRIS and CIDARS face challenges such as delays in early warning signals and limited data sources. Enhancing CIDARS through improved data integration and intelligent learning capabilities is necessary to increase the accuracy and timeliness of early warnings [37]. Table 3 shows the comparative table of China's notifiable disease surveillance systems.

**Table 3:** Comparative table of China's notifiable disease surveillance systems.

System	Indicator types	Timeliness	Spatial distribution	Surveillance capabilities
NNDSS	Reportable infectious diseases (A/B/C class); fundamental and longest history in China	Transitioned from paper-based monthly reporting to internet-based real-time reporting in 2004; covered all county-level CDCs by 2013	Nationwide coverage of all provinces, prefectures, counties; widest coverage among systems	Mainly case-reporting system, not automated warning; provides foundational data for research and policy
NIDRIS	Individual case data, laboratory-confirmed and ILI surveillance integrated for influenza	Real-time, mandatory reporting with high specificity	Nationwide, mandatory for all healthcare institutions	Enhances early detection of influenza epidemics, but lacks multi-sectoral data exchange; limited by data mainly from medical institutions
CIDARS	Aberration signals derived from NIDRIS data; covers 30+ reportable diseases by 2011	Real-time daily automated analysis; sends SMS alerts at 8 a.m. daily	Nationwide, all CDCs above county level by 2015	Automated multi-method warning (FDM, temporal and spatiotemporal models); alerts verified within 12 h; limitations in delayed checkpoints and limited data sources

Abbreviations: NNDSS, National Notifiable Diseases Surveillance System; NIDRIS, National Notifiable Infectious Diseases Reporting Information System; CIDARS, China Infectious Disease Automated-alert and Response System; ILI, influenza-like illness; CDC, center for disease control and prevention; SMS, Short Message Service; FDM, fixed-threshold detection method.

In recent years, the COVID-19 pandemic has further highlighted the limitations of relying solely on confirmed cases for timely warnings. In response, the National Intelligent Syndromic Surveillance System (NISSS) was proposed [43]. The system aims to use real-time information and existing knowledge to analyze suspected cases before clinical or laboratory confirmation, thereby achieving rapid control and early accurate prediction. The system is organized around a central data bank that continuously ingests multi-source inputs: case reports from hospital information systems/electronic health records and frontline clinicians; citizen crowdsourced submissions; cross-sector information systems; real-time data streams; internet activity data; and literature databases. These inputs are processed by Geographic Information System and AI-analytics modules to produce integrated situational assessments and early-warning signals that support decision-making. Alerts and information then move through a “reporting–verification/confirmation” chain across CDCs tiers, while feedback is provided to hospitals and clinicians and communications are disseminated to the public. Collectively, these steps enable coordinated surveillance and early warning grounded in real-time, multi-source data [43].

## 4 Discussion

### 4.1 The connection of environment-animal-human surveillance systems under the One Health perspective

**Fig. 2:**
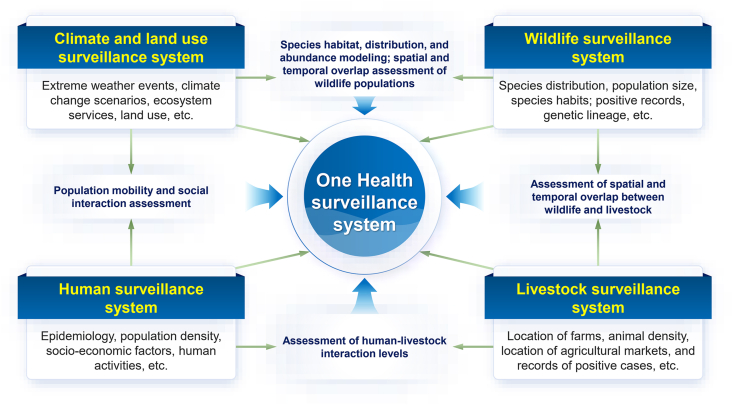
The framework of “One Health” surveillance proposed based on the environmental, animal and human surveillance systems.

One Health is a multi-sectoral approach designed to more effectively address complex health threats [12]. Surveillance systems under the One Health approach form a comprehensive framework, which is designed to systematically monitor “climate–animal–human” health [45,46]. This structural framework covers climate-driven ecological changes, early warning of animal epidemics, the capture of human health risks, and feedback adjustment of policies and interventions, forming a truly collaborative surveillance system [47]. Fig. 2 shows the framework of One Health surveillance proposed based on the environmental, animal, and human surveillance systems.

Within the framework of the One Health surveillance system, a central system serves as the hub, integrating four types of monitoring: climate and land use (extreme weather, climate scenarios, ecosystem services, land use, etc.); wildlife (distribution and population size, habits, positive cases, genetic lineage, etc.); livestock (farm and market locations, animal density, positive case records, etc.); and human populations (epidemiology, population density, socioeconomic factors, human activities, etc.). These four systems work together to achieve One Health surveillance: climate and land use changes influence wildlife habitat and distribution patterns, and affect human mobility and social interactions; spatial and temporal overlap between wildlife and livestock reveals potential spillover pathways; and the intensity of human–livestock contact and positive test results characterize zoonotic disease transmission risks. The One Health surveillance system organically connects climate–animals–human relationships, and by continuously monitoring climate anomalies or ecological environmental changes (such as precipitation, temperature, vegetation cover, etc.), potential pathogen signals in animals can be discovered in a timely manner. When the human health monitoring system detects unusual cases or the risk of an outbreak, it can quickly trigger public health interventions.

### 4.2 Shortcomings and prospects of China's national surveillance system under the One Health perspective

The concept of One Health emphasizes the integrated nature of human, animal, and environmental health, highlighting their interactions. China's national-level surveillance system is an important component of the country's strategy to safeguard public health amid increasingly complex and interconnected environmental and biological risks. The integration of climate, wildlife and livestock, and human disease surveillance within the One Health framework, enables a holistic approach to managing public health risks, particularly those arising from zoonotic diseases and environmental change. However, China's national surveillance system still has some shortcomings.

First, from the perspective of surveillance data, data coverage and collection accuracy require improvement, especially in remote areas and emerging fields where outdated monitoring equipment and technology result in incomplete or inaccurate information [26]. The mechanisms for data sharing and integration are underdeveloped, and information barriers between departments still exist, hindering the overall monitoring efficiency and timeliness of decision-making [48].

Second, from the perspective of monitoring and early warning technology, real-time monitoring and early warning capabilities are insufficient, and the system's response speed and coordination capabilities remain limited in emergency situations [49]. In addition, meteorological, animal, and human health monitoring mostly relies on independent technology platforms, lacking effective integration and intelligent early warning capabilities. This is especially true in remote areas, where the real-time capacity and accuracy of monitoring data are limited [50].

Finally, from the perspective of policy coordination, deficiencies in professional training and technological innovation constrain the ongoing improvement and modernization of the system [51]. Furthermore, the transparency and public participation of the monitoring system is low, and information disclosure is insufficient, affecting public trust and collaboration [52]. To address these challenges, the effectiveness and reliability of China's national surveillance system should be comprehensively enhanced through increased investment, institutional reform, the improvement of cross-sectoral cooperation, and the promotion of technological advancement.

To address the limitations of the current surveillance system within the One Health framework, a range of initiatives has been undertaken in China [53,54]. (1) Enhancing multi-source surveillance capacity: Through optimizing current infectious disease reporting systems, the country aims to establish a comprehensive online reporting system by all healthcare institutions by 2030. This will include the clinical syndromes-based nationwide sentinel surveillance hospitals and laboratory-based pathogen surveillance across multiple sectors and institutions. The surveillance network will include vectors, host animals, and environmental risk factors, with enhanced cross-sectoral coordination involving education, civil affairs, agriculture, customs, and environmental protection. (2) Standardizing early warning mechanisms and advancing digital intelligent forecasting: A national-level integrated platform for disease surveillance, early warning, and emergency command is in development. The early warning protocols are being refined to include information dissemination standards and emergency response procedures, which will enable timely public communication and coordinated interventions. In parallel, enhancing data governance and sharing capabilities, cybersecurity and computing power support and developing models for anomaly detection, outbreak prediction, and decision support based on big data, AI, and cloud computing, can create a favorable environment for the effective implementation of the One Health approach. (3) Improving human and technical capacity: The interdisciplinary expert teams and committees related to animal and human health are being established. Laboratory capacity is being expanded to ensure Biosafety Level (BSL)-3 laboratories at the provincial level and BSL-2 coverage at the city level, with high-throughput, multi-pathogen detection capabilities and emergency diagnostic tools. The workforce in hospital and public health institution labs and the field is prioritized for appropriate training. Moreover, it should develop an interoperable One Health in-service training course for people from environmental, healthcare, and veterinary sectors. (4) Reinforcing governance, financing, and international cooperation: Government leadership is being strengthened by incorporating surveillance into key public agendas and clarifying responsibility across local governments, institutions, and individuals. Financial support mechanisms prioritize high-risk regions and critical infrastructure. Scientific research on pathogen detection, data governance, and intelligent forecasting is promoted through national innovation programs. International collaboration is also encouraged via data sharing, joint surveillance, and personnel exchanges with global and regional partners. These comprehensive efforts aim to build a resilient, responsive, and integrated surveillance system aligned with the One Health approach, thereby enhancing China's ability to prevent and control emerging disease threats to public health.

## 5 Conclusions

China has established surveillance systems encompassing climate monitoring, wildlife health, livestock management, and population health. The One Health approach, which emphasizes the interconnection between human, animal, and environmental health, offers a comprehensive framework to enhance the effectiveness and reliability of these national monitoring systems. To this end, several strategic measures should be considered: enhancing multi-source surveillance capacity; standardizing early warning mechanisms and advancing digital intelligent forecasting; building human and technical capacity; and reinforcing governance, financing, and international cooperation. These efforts will facilitate better integration and data sharing across surveillance systems, strengthening China's capacity to detect, monitor and respond to emerging and re-emerging infectious diseases at an early stage, thereby safeguarding public health and safety.

## CRediT authorship contribution statement

**Zhichao Li:** Writing – review & editing, Writing – original draft, Methodology, Funding acquisition. **Dongliang Li:** Writing – review & editing, Writing – original draft, Methodology. **Jinwei Dong:** Writing – review & editing, Writing – original draft, Supervision. **Qixu Zhu:** Writing – review & editing. **Youyi Zuo:** Writing – review & editing, Writing – original draft. **Juan Pu:** Writing – review & editing, Writing – original draft. **Lu Wang:** Writing – review & editing, Writing – original draft. **Weipan Lei:** Writing – review & editing, Writing – original draft. **Jun Cai:** Writing – review & editing, Writing – original draft. **Qu Cheng:** Writing – review & editing, Writing – original draft. **Yuzhe Li:** Writing – review & editing, Writing – original draft. **Jing Yang:** Writing – review & editing, Writing – original draft. **Yang Ju:** Writing – review & editing, Writing – original draft. **Zhirui Wu:** Writing – review & editing, Writing – original draft.

## Funding

This research was funded by the 10.13039/501100012166National Key Research and Development Program of China (grant number 2022YFF0802400), and the Science and Technology Innovation Action Plan of Shanghai (grant number 24J22800900).

## Declaration of competing interest

The authors declare no competing interests.
